# The Effects of Experiencing Child Maltreatment and Sociodemographic Factors on Parental Conflict Tactics in Spain

**DOI:** 10.1177/13591045261421737

**Published:** 2026-01-28

**Authors:** Danilo Dominguez, Carles Pérez-Testor, Paula Benedico-Peydró, Berta Aznar-Martínez

**Affiliations:** 1 Universitat Ramon Llull, Faculty of Psychology, Education and Sport Sciences, Blanquerna, Barcelona, Spain

**Keywords:** child maltreatment, intergenerational violence, parental conflict tactics, non-violent discipline, coercive discipline, physical aggression

## Abstract

The intergenerational perpetuation of violence is one of the most concerning consequences of child maltreatment from both psychological and social perspectives. This study examined the presence of the cycle of violence within the Spanish context, understood as the transmission of aggressive parental conflict tactics to their children. The sample consisted of 700 mothers and fathers residing in Spain, all of whom had at least one child under the age of 18. Using standardized measures of childhood maltreatment and parental conflict tactics, the findings confirmed the persistence of the cycle of violence, particularly for individuals who had experienced physical abuse, sexual abuse, or physical neglect in childhood. Significant associations were also observed between childhood maltreatment and certain sociodemographic characteristics, suggesting that both early adverse experiences and contextual factors contribute to the use of coercive or aggressive parental conflict tactics. A comprehensive understanding of these dynamics is essential for designing effective prevention strategies, informing clinical interventions, and developing public policies aimed at reducing child maltreatment and preventing its transmission across generations.

## Plain Language Summary

Childhood Maltreatment, Social Factors, and Parental Conflict Tactics in Spain: The passing of violence from one generation to the next is one of the most worrying consequences of child maltreatment, both psychologically and socially. This study looked at whether this “cycle of violence” exists in Spain. The cycle was understood as the way aggressive ways of handling conflict between parents are learned in childhood and later repeated in adult life. The study included 700 mothers and fathers living in Spain, all of whom had at least one child under the age of 18. Using standardized questionnaires about childhood maltreatment and how parents manage conflicts, the results confirmed that the cycle of violence continues to exist. This was especially true for people who had experienced physical abuse, sexual abuse, or physical neglect during childhood. The study also found important links between childhood maltreatment and certain sociodemographic factors. This suggests that both early negative experiences and social context influence the use of aggressive or controlling behaviors in parental conflicts. Understanding these processes is essential for developing effective prevention strategies, improving clinical interventions, and creating public policies that reduce child maltreatment and prevent violence from being passed on to future generations.

## Introduction

Child maltreatment encompasses various forms of psychological, physical, and sexual abuse, as well as emotional and physical neglect, directed at individuals under the age of 18 (World Health Organization [WHO], 2024; UNICEF, 2025). This form of violence can have a significant impact on an individual’s psychological, neurological, physical, and social development, as well as their functioning, particularly influencing their experiences in adulthood (Boynton-Jarrett et al., 2021; Ciurria, 2018; Sahle et al., 2022; Warmingham et al., 2021).

Numerous studies have demonstrated a relationship between childhood maltreatment and various psychological and behavioral outcomes, including resilience capacity, attachment style, and personality traits (Arfeen & Dangwal, 2024; Boland et al., 2020; Dominguez et al., 2025a), as well as suicidal behavior (Angelakis et al., 2020), substance abuse, emotional dysregulation, and negative emotional processing. These latter factors are often associated with the development of affective disorders such as depression (Bounoua & Sadeh, 2022; Islam et al., 2022; Tingtin et al., 2021; UNICEF, 2025; WHO, 2024). In general, women are more likely to experience internalizing problems, such as depression, anxiety, post-traumatic stress disorder, and suicidal ideation or attempts, while men tend to exhibit a higher prevalence of externalizing problems, including disruptive behavior, substance use, and violent or delinquent conduct (Martín-Ruiz et al., 2024; Rey et al., 2023; UNICEF, 2025).

One of the most concerning aspects of child maltreatment from both psychological and social perspectives is the perpetuation of violence across generations, which leads to new victims among the next generation of children. Research has shown the existence of a cycle of violence, wherein parental conflict tactics are often repeated. Parents with a family history of maltreatment, neglect, and dysfunctional family environments are more likely to replicate these forms of violence with their own children (Hellmann et al., 2018; Save the Children, 2018; WHO, 2024). In addition to punitive parental conflict tactics and a history of child maltreatment, several family characteristics have been identified as significant risk factors for child maltreatment. These include low parental education, economic hardship, unemployment, large family size, and belonging to ethnic minorities (particularly non-European or mixed ethnic backgrounds), among other contextual and structural factors (Ahn et al., 2022; Clemens et al., 2020; Save the Children, 2018; WHO, 2022; UNICEF, 2025).

In the Netherlands, Kong et al. (2021) conducted a study to examine whether experiencing childhood maltreatment was associated with violence against their own children. The results indicated a more frequent use of emotional abuse, physical abuse, and neglectful abuses towards children among parents who reported a history of childhood maltreatment. In Pakistan, Lakhdir et al. (2019) concluded that emotional abuse increased in families where parents had a history of childhood victimization. However, in China, Hayes et al. (2020) demonstrated that, although the majority of the sample had experienced childhood maltreatment (64.10%), there was no association with attitudes towards domestic violence.

Undoubtedly, these discrepancies can be attributed to social and cultural differences, as the acceptance of certain parental conflict tactics, such as corporal punishment, may vary according to the cultural norms and values of each country or society (Dominguez et al., 2025b; Ranghetti & Milani, 2021). Furthermore, it is important to highlight the lack of accurate data on the actual number of children who experience maltreatment or other forms of victimization by their caregivers, including physical aggression, as many cases go unreported or are inadequately documented (Dominguez et al., 2025a, 2025b; UNICEF, 2025). In this context, contemporary society still lacks full awareness that certain parental conflict tactics involving shouting, insults, or physical aggression constitute forms of violence with serious physical and emotional consequences for children (Dominguez et al., 2025a; Save the Children, 2018). Thus, although some authors consider a history of childhood maltreatment to be one of the main risk factors for engaging in parental violence, and thereby repeating the cycle of violence with one’s own children, discrepancies in this regard have also been observed (Hellmann et al., 2018).

In Spain, the existing literature supports the notion of intergenerational violence; however, most studies conducted within the Spanish context have focused on specific populations rather than on the broader transmission of childhood maltreatment into parental conflict tactics. In particular, a substantial body of research has examined children exposed to gender-based violence, analyzing the psychological, emotional, and behavioral consequences of growing up in households where parental figures perpetrate violence against one another (Alvarado Castillo, 2025; Moya & Rodríguez, 2025). Another significant group of studies has centered on child-to-parent violence—that is, aggression directed by children toward their caregivers (Cuervo García & Gracia, 2021; Selma, 2022). Although this phenomenon is often interpreted through an intergenerational lens —such as the internalization and reproduction of violent models learned within the family— these studies do not directly address how adults may reproduce parental conflict tactics shaped by the maltreatment they experienced in their own childhood. In sum, the available evidence demonstrates that intergenerational violence is a relevant phenomenon in Spain; however, it also reveals a substantial gap: the absence of studies specifically examining the relationship between childhood maltreatment experiences and the subsequent use of parental conflict tactics in adulthood. This conceptual and empirical gap underscores the need for studies such as the present one, which aim to broaden the understanding of the cycle of violence within the Spanish context.

The main purpose of this study is to investigate the presence of the cycle of violence within the Spanish context, understood as the intergenerational transmission of violent behavior from parents to children. This phenomenon holds significant social and clinical relevance due to its impact on perpetuating dysfunctional family dynamics and the well-being of future generations. To address this overarching aim, the following specific objectives have been established:

(1) To analyze the associations between different types of childhood maltreatment (emotional abuse, physical abuse, sexual abuse, emotional neglect, and physical neglect) and the use of parental conflict tactics (non-violent discipline, coercive discipline, and physical aggression).
  (a) **Hypothesis 1:** Experiencing any type of childhood maltreatment is associated with increased use of more aggressive parental conflict tactics (coercive discipline and physical aggression), while no notable associations are expected regarding non-violent discipline
(2) To examine the individual effects of each type of childhood maltreatment on the use of the parental conflict tactics, assessing the specific impact of each maltreatment experience on parental conflict tactics.
  (a) **Hypothesis 2:** Each type of childhood maltreatment is directly related to the use of more aggressive parental conflict tactics (coercive discipline and physical aggression), while no notable effects are expected for non-violent discipline.
(3) To evaluate the combined impact of the different types of childhood maltreatment on the parental conflict tactics, identifying the most significant predictors when considered simultaneously, and controlling for the effects of other forms of maltreatment.
  (a) **Hypothesis 3:** When the different types of childhood maltreatment are considered simultaneously, certain types of abuse, particularly emotional, physical, and sexual abuse, are expected to be the most significant predictors of more aggressive parental conflict tactics (coercive discipline and physical aggression), while no notable effects are expected regarding non-violent discipline.

Moreover, the present study seeks to examine additional variables that may contribute to the dynamics of the cycle of violence:

(4) To analyze the influence of sociodemographic variables (sex, age, marital status, substance use, ethnic group, religious affiliation, educational level, employment status, income, and family structure) on the use of parental conflict tactics, controlling for childhood maltreatment experiences as an independent variable.
  (a) **Hypothesis 4:** Specific sociodemographic variables —particularly sex, educational level, employment status, income, alcohol and drug use, and family structure— will be associated with the use of parental conflict tactics, even after controlling for childhood maltreatment experiences.

## Method

### Data Collection

The data were collected by a company specialized in the distribution, collection, and analysis of information, using a quantitative approach based on the CAWI (Computer Aided Web Interviewing) method applied to an online panel. Sampling was carried out through a quota-based stratified system by sex, with fixed quotas of 50% men and 50% women to ensure the representativeness of the overall sample. Inclusion criteria required participants to be residents of Spain, specifically the autonomous community of Catalonia, adults, parents, and to have at least one child under 18 years of age.

The data collection procedure was characterized by rigorous quality-control measures designed to ensure the integrity and reliability of the information obtained. A minimum reasonable completion time was estimated, and all responses falling below this threshold were removed. Attention-check items were included to assess participant attentiveness, and internal response consistency was examined to identify potential inconsistencies. Surveys exhibiting contradictory answers or unreliable response patterns were discarded. Clear instructions were provided throughout the questionnaire to facilitate accurate comprehension of each item. These procedures, together with automated fraud-detection systems, ensured a precise and reliable data collection process.

Regarding ethical considerations, participants were presented with a brief description of the study before beginning the survey, informing them of the research objectives and the confidential nature of their responses. The collection, processing, storage, communication, and transfer of data were carried out in accordance with the General Data Protection Regulation (EU Regulation 2016/679 of the European Parliament and of the Council of 27 April 2016) and with the applicable Spanish data protection laws, including Royal Decree 1720/2007, which develops Organic Law 15/1999, and Law 41/2002 on patient autonomy and clinical information rights. The study’s ethical aspects were reviewed and approved by the Ethics and Research Committee of the Faculty of Psychology, Education and Sport Science, Blanquerna, Ramon Llull University, with code 2122016D.

Because the questionnaire included potentially sensitive content, such as experiences of child maltreatment, the possibility of emotional discomfort or the reactivation of past traumatic experiences was considered. For this reason, the introductory section of the questionnaire included a warning about the nature of the content and reminded participants that their participation was fully voluntary and that they could withdraw at any time without consequence. Contact information for psychological support services was provided, as well as the research team’s email address for any questions or needs related to the study. All members of the research team were psychologists specialized in violence, ensuring an ethical, sensitive, and professional approach to the topics addressed.

### Participants

The present study was conducted in Spain, specifically in the autonomous community of Catalonia, with a sample of 700 adult mothers and fathers (*M* = 41.23; *SD* = 8.30), evenly distributed by sex (350 women; 350 men), all of whom had at least one minor child (*M* = 9.85; *SD* = 4.82). For a more detailed description of the sample’s sociodemographic characteristics, Table 1 presents an expanded version of a table previously published in Dominguez et al. (2025a), now including additional data on participants’ alcohol and drug use.

**Table 1.** Sociodemographic Data

	Man n = 350 (50%)		Woman n = 350 (50%)		Total n = 700 (100%)	
Marital status						
Married	237	(56.2%)	185	(43.8%)	422	(60.3%)
Coupled	79	(48.2%)	85	(51.8%)	164	(23.4%)
Single	15	(25%)	45	(75%)	60	(8.6%)
Divorced	16	(33.3%)	32	(66.7%)	48	(6.9%)
Widowed	3	(50%)	3	(50%)	6	(0.9%)
Educational level						
Elementary	2	(50%)	2	(50%)	4	(0.6%)
High school (12-16 yrs)	54	(50.5%)	53	(49.5%)	107	(15.3%)
High school (16-18 yrs)	55	(56.1%)	43	(43.9%)	98	(14%)
Inter. vocational training	42	(46.2%)	49	(53.8%)	91	(13%)
Adv. vocational training	53	(42.1%)	73	(57.9%)	126	(18%)
University studies	97	(48%)	105	(52%)	202	(29.9%)
Master	40	(62.5%)	24	(37.5%)	64	(9.1%)
PhD	7	(87.5%)	1	(12.5%)	8	(1.1%)
Employment status						
Employed	287	(52.9%)	256	(47.1%)	543	(77.6%)
Unemployed	23	(41.8%)	32	(58.2%)	55	(7.9%)
Self-employed	22	(51.2%)	21	(48.8%)	43	(6.1%)
Homemaker	2	(8.7%)	21	(91.3%)	23	(3.3%)
Retired	2	(28.6%)	5	(71.4%)	16	(2.3%)
Unable to work	6	(46.2%)	7	(53.8%)	13	(1.9%)
Student	4	(57.1%)	3	(42.9%)	7	(1%)
Annual salary						
No annual income	13	(29.6%)	31	(70.4%)	44	(6.3%)
Very low level income	20	(22.2%)	70	(77.8%)	90	(12.9%)
Low level income	57	(38%)	93	(62%)	150	(21.4%)
Mid level income	124	(57.1%)	93	(42.9%)	217	(31%)
High level income	96	(67.1%)	47	(32.9%)	143	(20.4%)
Very high level income	40	(71.4%)	16	(28.6%)	56	(8.1%)
Nuclear family						
Lives alone	6	(85.7%)	1	(14.3%)	7	(1%)
Lives with 1 person	8	(21.6%)	29	(78.4%)	37	(5.3%)
Lives with 2 people	152	(52.2%)	139	(47.8%)	291	(41.6%)
Lives with 3 people	146	(52.5%)	132	(47.5%)	278	(39.7%)
Lives with 4 or more people	38	(43.7%)	49	(56.3%)	87	(12.4%)
Ethnic group						
Caucasian	284	(50.8%)	275	(49.2%)	559	(79.9%)
Latin American	45	(44.6%)	56	(55.4%)	101	(14.4%)
Middle Eastern	12	(42.9%)	16	(57.1%)	28	(4%)
Afrodescendant	9	(75%)	3	(25%)	12	(1.7%)
Religious affiliation						
Christianity	197	(50.5%)	193	(49.5%)	390	(55.7%)
Atheism	78	(43.6%)	101	(56.4%)	179	(25.6%)
Agnosticism	52	(54.2%)	44	(45.8%)	96	(13.7%)
Other affiliations	23	(65.7%)	12	(34.3%)	35	(4.9%)
Alcohol						
No consumption	47	(41.23%)	67	(58.77%)	114	(16.29%)
Occasionally	100	(41.32%)	142	(58.68%)	242	(34.57%)
From time to time	126	(56.25%)	98	(43.75%)	224	(32%)
Frequently	77	(64.17%)	43	(35.83%)	120	(17.14%)
Drugs						
No use	302	(48.48%)	321	(51.52%)	623	(89%)
Use	48	(62.34%)	29	(37.66%)	77	(11%)

*Note.* Adapted from Dominguez et al. (2025a). This version includes additional data on alcohol and drug use not included in the original publication.

### Instruments

#### Conflict Tactics Scale: Parent to Child (CTSPC)

The *Conflict Tactics Scale: Parent to Child* (CTSPC) (Straus et al., 1998) - Spanish version (Dominguez et al., 2025b) measures the tactics or behavior by parents when facing conflicts or expressing hostility towards their children. Thus, the Spanish version of the CTSPC consists of a total of 21 items distributed across 3 dimensions: Non-Violent Discipline (NVD; α = .71), which refers to disciplinary practices in which no violence is exerted; Coercive Discipline (CD; α = .81), which captures the interaction between emotional abuse and low-intensity physical aggression, which, although not reaching the levels of severe violence, can still have a significant negative impact on the child’s development; and Physical Aggression (PAG; α = .93), which encompasses extremely severe forms of aggression, such as administering beatings.

#### Child Trauma Questionnaire (CTQ) – Short Form

The *Childhood Trauma Questionnaire -short form* (CTQ-SF; Bernstein & Fink, 1998) - Spanish version (Hernández et al., 2012) is made up of 28 items (25 clinical items and 3 validity items), answered on a 5-point Likert-type scale (0 = Never; 5 = Very often), which examine a total of five types of child abuse: emotional abuse (EA; α = 0.84), physical abuse (PA; α = 0.88), sexual abuse (SA; α = 0.94), emotional neglect (EN; α = 0.83), and physical neglect (PN; α = 0.65).

#### Ad hoc Sociodemographic Questionnaire

The participants provided information about sex (man/woman), age (numerical response), marital status (married, coupled, single, divorced, widowed), educational level (elementary education, high school [12–16 years], high school [16–18 years], intermediate vocational training, advanced vocational training, university studies, master’s degree, PhD), employment status (employed, unemployed, self-employed, homemaker, retired, unable to work, student), annual net income adapted to the Spanish context (no annual income, very low level income [under 12.450 euros], low level income [12.451–20.200 euros], mid level income [20.201–35.200], high level income [35.201–60.000 euros], very high level income [60.001 euros or more]), nuclear family (lives alone, lives with 1 person, lives with 2 people, lives with 3 people, lives with 4 or more people), ethnic group (Caucasian, Latin American, Middle Eastern, Afrodescendant), religious affiliation (christianity, atheism, agnosticism, other affiliations), frequency of alcohol consumption (no consumption, occasionally, from time to time, frequently), and drug use (no use, use). For more details, see Table 1.

### Data Analysis

All analyses were conducted using the statistical software SPSS (v.26). Descriptive statistics, including frequencies, percentages, means, and standard deviations, were calculated to characterize the sociodemographic profile of the sample.

For the first block, which addressed the intergenerational cycle of violence, analyses were conducted according to the study objectives:

#### Objective 1

This objective examined general associations between variables, without explicitly distinguishing between dependent and independent variables. Spearman correlation analyses were conducted to explore the relationships between different types of childhood maltreatment (emotional abuse, physical abuse, sexual abuse, emotional neglect, and physical neglect) and parental conflict tactics with children (non-violent discipline, coercive discipline, and physical aggression). Spearman’s rho was used due to the non-normal distribution of the study variables.

#### Objective 2

The dependent variable was the use of parental conflict tactics, and the independent variables were the five types of childhood maltreatment. Simple linear regression analyses were conducted to evaluate the individual effects of each type of maltreatment on parental conflict tactics.

#### Objective 3

Once again, the dependent variable was the use of parental conflict tactics, and the independent variables were the five types of childhood maltreatment. Multiple linear regression analyses were performed, simultaneously including all five types of maltreatment as predictors, in order to identify the most significant effects while controlling for shared variance among the different maltreatment types. Assumptions of collinearity were assessed using tolerance indices and the Variance Inflation Factor (VIF). Tolerance values remained above .20 and VIF values below 5, indicating the absence of problematic multicollinearity.

In the second block of analyses, the influence of sociodemographic variables (sex, age, marital status, substance use, ethnic group, religious affiliation, educational level, employment status, income, and family structure) on parental conflict tactics was examined, controlling for childhood maltreatment experiences.

#### Objective 4

Parental conflict tactics remained the dependent variable, while sociodemographic factors served as independent variables, with childhood maltreatment included as a control variable. Hierarchical regression models were applied, entering childhood maltreatment variables in the first step, followed by sociodemographic variables in the second step, to assess the additional contribution of sociodemographic factors to the use of parental conflict tactics.

## Results

### Description of the Study Sample

A total of 24.4% of the parents in the sample reported having been victims of psychological, physical, and/or sexual abuse during childhood. Specifically, 20.5% reported having experienced emotional abuse, 12.1% physical abuse, and 6.4% sexual abuse (Dominguez et al., 2025a). Regarding parental conflict tactics used with their children, between 64.86% and 88.57% of participants reported primarily employing non-violent discipline (NVD). To a lesser extent, the use of coercive discipline (CD) was reported by 6.57% to 25.86% of participants, while between 1% and 6.43% acknowledged using physical aggression (PAG) as part of their parental conflict tactics. In addition, 14.14% of participants have used physical aggression towards their children at least once in their lifetime. For a more detailed description of the sample’s sociodemographic characteristics, see Table 1.

### Relationship Between Childhood Maltreatment Experiences and Parental Conflict Tactics

To address Hypothesis 1, bivariate correlations were conducted to examine the associations between the different types of childhood maltreatment and parental conflict tactics. As shown in Table 2, non-violent discipline was significantly associated only with emotional abuse. In contrast, both coercive discipline and physical aggression showed positive and significant associations with all forms of childhood maltreatment assessed. However, effect sizes were generally small, indicating that although these relationships are consistent, their magnitude is limited. In parallel, the different types of childhood maltreatment were significantly correlated with one another, with coefficients ranging from moderate to high. This pattern reflects a notable interdependence among maltreatment forms, which is particularly relevant for the subsequent analyses.

**Table 2.** Spearman’s Correlations Among Childhood Maltreatment and Parental Conflict Tactics

		Emotional abuse	Physical abuse	Sexual abuse	Emotional neglect	Physical neglect
Emotional abuse	r	-				
	*p*	-				
Physical abuse	r	.613	-			
	*p*	<.001	-			
Sexual abuse	r	.419	.449	-		
	*p*	<.001	<.001	-		
Emotional neglect	r	.607	.488	.295	-	
	*p*	<.001	<.001	<.001	-	
Physical neglect	r	.532	.481	.335	.583	-
	*p*	<.001	<.001	<.001	<.001	-
Non-violent discipline	r	**.157**	.028	−.034	.063	−.026
	*p*	**< .001**	.463	.370	.097	.494
Coercive discipline	r	**.260**	**.231**	**.125**	**.178**	**.157**
	*p*	**< .001**	**< .001**	**< .001**	**< .001**	**< .001**
Physical aggression	r	**.127**	**.201**	**.173**	**.100**	**.132**
	*p*	**< .001**	**< .001**	**< .001**	**.008**	**< .001**

*Note.* Spearman correlation coefficients indicate both the direction and magnitude of the association, with r = .10 considered small, r = .30 medium, and r = .50 large (Cohen, 1988). Bold indicate statistically significant results (*p* < 0.05).

To address Hypothesis 2, simple linear regressions were conducted to evaluate the individual effect of each type of childhood maltreatment on parental conflict tactics. As shown in Table 3, for non-violent discipline, only emotional abuse (R^2^ = 2.2%) and emotional neglect (R^2^ = 0.6%) emerged as significant predictors; physical abuse, sexual abuse, and physical neglect did not show significant effects (*p* > .05). For coercive discipline, all forms of maltreatment were significant predictors: emotional abuse (R^2^ = 4.6%), physical abuse (R^2^ = 5.6%), sexual abuse (R^2^ = 3.5%), emotional neglect (R^2^ = 2.2%), and physical neglect (R^2^ = 2.5%).

**Table 3.** Individual Effects of Different Types of Child Maltreatment on Parental Conflict Tactics

Parental conflict tactics	Childhood maltreatment	B	β	IC 95%	R^2^	F (1, 699)	*p*
Non-violent discipline	Emotional abuse	0.841	0.150	[0.429, 1.253]	.022	16.041	**<.001**
	Physical abuse	0.468	0.060	[−0.113, 1.049]	.004	2.498	.114
	Sexual abuse	0.119	0.016	[−0.447, 0.685]	.000	0.171	.680
	Emotional neglect	0.434	0.077	[0.014, 0.853]	.006	4.110	**.043**
	Physical neglect	0.051	0.006	[−0.553, 0.655]	.000	0.027	.869
Coercive discipline	Emotional abuse	0.595	0.216	[0.395, 0.796]	.046	34.006	**<.001**
	Physical abuse	0.909	0.236	[0.630, 1.187]	.056	41.016	**<.001**
	Sexual abuse	0.701	0.187	[0.428, 0.975]	.035	25.334	**<.001**
	Emotional neglect	0.418	0.150	[0.213, 0.623]	.022	16.008	**<.001**
	Physical neglect	0.633	0.158	[0.340, 0.927]	.025	17.928	**<.001**
Physical aggression	Emotional abuse	0.314	0.171	[0.179, 0.449]	.029	20.919	**<.001**
	Physical abuse	0.759	0.295	[0.576, 0.942]	.087	66.489	**<.001**
	Sexual abuse	0.762	0.305	[0.585, 0.939]	.093	71.451	**<.001**
	Emotional neglect	0.072	0.038	[−0.067, 0.210]	.001	1.032	.310
	Physical neglect	0.609	0.228	[0.416, 0.802]	.052	38.300	**<.001**

Bold indicate statistically significant results (*p* < 0.05).

Regarding physical aggression, four types of maltreatment were significant predictors: emotional abuse (R^2^ = 2.9%), physical abuse (R^2^ = 8.7%), sexual abuse (R^2^ = 9.3%), and physical neglect (R^2^ = 5.2%), whereas emotional neglect did not show a significant effect (*p* > .05). Overall, the proportions of explained variance (R^2^) ranged from low to moderate, indicating that each type of maltreatment accounts for only a limited portion of the variability in parental conflict tactics. All unstandardized coefficients (B), standardized coefficients (β), and 95% confidence intervals are presented in Table 3. These findings informed the subsequent multiple regression analyses.

To address Hypothesis 3, a multiple linear regression analysis was conducted to simultaneously evaluate the impact of the different forms of childhood maltreatment on parental conflict tactics.

First, regarding non-violent discipline, the multiple regression model was statistically significant, F (5, 699) = 5.119, *p* < .001, explaining 3.6% of the variance (R^2^ = .036). However, emotional abuse was the only significant predictor, while the remaining forms of maltreatment did not reach statistical significance (*p* > .05).

Second, for coercive discipline, the multiple regression model was also statistically significant, F (5, 699) = 9.851, *p* < .001, accounting for 6.6% of the variance (R^2^ = .066). The significant predictors were physical abuse and sexual abuse, whereas emotional abuse, emotional neglect, and physical neglect did not show significant effects (*p* > .05).

Third, with respect to physical aggression, the multiple regression model was again statistically significant, F (5, 699) = 24.05, *p* < .001, explaining 14.8% of the variance (R^2^ = .148). Significant predictors included physical abuse, sexual abuse, physical neglect, and emotional neglect, while emotional abuse was not a significant predictor (*p* > .05).

#### Influence of Sociodemographic Variables on Parental Conflict Tactics

**Figure 1.**
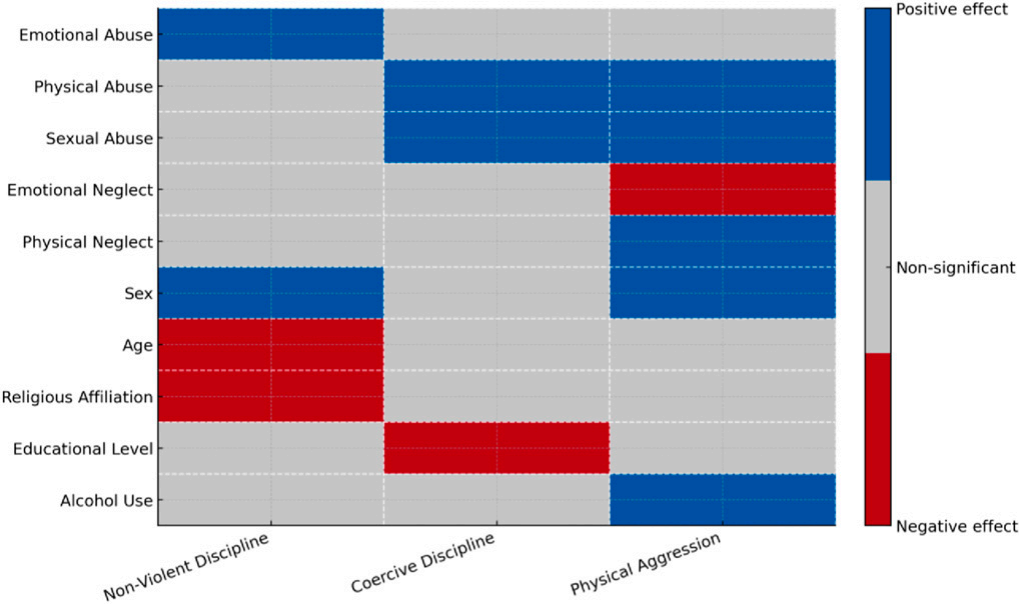
Significant predictors of parental conflict tactics. *Note*: This figure was generated using artificial intelligence (AI). Blue cells indicate significant positive effects; red cells indicate significant negative effects; and grey cells represent non-significant associations. Predictors include the five forms of childhood maltreatment (emotional abuse, physical abuse, sexual abuse, emotional neglect, and physical neglect) and five sociodemographic variables (sex, age, religious affiliation, educational level, and alcohol use). Outcomes correspond to the three parental conflict tactics assessed in the study: non-violent discipline, coercive discipline, and physical aggression

To address Hypothesis 4, hierarchical regression analyses were conducted to examine the influence of sociodemographic variables on parental conflict tactics, while controlling for the effects of childhood maltreatment. Block 1 —which included the five types of childhood maltreatment (emotional abuse, physical abuse, sexual abuse, emotional neglect, and physical neglect)— was presented previously in Table 4, where their effects on each parental tactic were detailed. Therefore, the present section reports only the results from Block 2, which incorporated sociodemographic variables (sex, age, marital status, educational level, employment status, annual net income, family structure, ethnic group, religious affiliation, frequency of alcohol use, and drug use) in order to assess their additional contribution to the prediction of parental conflict tactics once childhood maltreatment had been controlled for. In Figure 1, a visual synthesis of all results is presented, showing in an integrated manner which types of childhood maltreatment and which sociodemographic variables predict each of the parental conflict tactics.

**Table 4.** Multiple Effects of Different Types of Child Maltreatment on Parental Conflict Tactics

Parental conflict tactic	Predictor	B	β	95% CI	*p*
Non-violent discipline	Emotional abuse	1.560	0.278	[0.837, 2.282]	**<.001**
	Physical abuse	−0.340	−0.043	[−1.206, 0.526]	.441
	Sexual abuse	−0.287	−0.038	[−0.943, 0.369]	.391
	Emotional neglect	−0.169	−0.030	[−0.779, 0.441]	.586
	Physical neglect	−0.814	−0.100	[−1.643, 0.015]	.054
Coercive discipline	Emotional abuse	0.210	0.076	[−0.140, 0.560]	.239
	Physical abuse	0.566	0.147	[0.147, 0.985]	**.008**
	Sexual abuse	0.336	0.090	[0.019, 0.654]	**.038**
	Emotional neglect	0.032	0.012	[−0.263, 0.328]	.831
	Physical neglect	−0.081	−0.020	[−0.483, 0.320]	.691
Physical aggression	Emotional abuse	−0.069	−0.037	[−0.292, 0.154]	.545
	Physical abuse	0.615	0.239	[0.348, 0.882]	**<.001**
	Sexual abuse	0.521	0.209	[0.319, 0.724]	**<.001**
	Emotional neglect	−0.329	−0.177	[−0.517, −0.141]	**<.001**
	Physical neglect	0.314	0.118	[0.058, 0.570]	**.016**

Bold indicate statistically significant results (*p* < 0.05).

In the first model, with non-violent discipline as the dependent variable, Block 1 (childhood maltreatment) explained only a minimal proportion of variance, consistent with previous analyses. When Block 2 (sociodemographic variables) was added, the model reached statistical significance, F (11, 688) = 2.957, p < .001, accounting for 4.5% of the total variance (R^2^ = .045). Three sociodemographic variables emerged as significant predictors: sex, age, and religious affiliation. Women (M = 29.56, SD = 24.54) scored higher on non-violent discipline than men (M = 24.55, SD = 23.63); age was negatively associated with non-violent discipline, indicating that older participants used this strategy less frequently; and religious affiliation showed that atheists (M = 31.37, SD = 24.81) reported higher use of non-violent discipline than agnostics (M = 25.16, SD = 23.32). The remaining variables did not reach statistical significance (ps > .05). Complete coefficients for Block 2 are presented in Table 5.

**Table 5.** Hierarchical Regression Model Predicting Non-Violent Discipline From Sociodemographic Variables (Block 2) Controlling for Childhood Maltreatment

Predictor	B	β	95% CI	*p*
Sex	4.648	–	[0.855, 8.442]	**.016**
Age	−0.260	−0.089	[−0.485, −0.034]	**.024**
Marital status	−1.625	−0.051	[−4.044, 0.794]	.188
Educational level	−0.198	−0.014	[−1.377, 0.981]	.742
Employment status	−1.112	−0.066	[−2.430, 0.206]	.098
Annual net income	−1.103	−0.060	[−2.782, 0.575]	.197
Nuclear family	1.529	0.051	[−0.669, 3.728]	.172
Ethnic group	−2.612	−0.075	[−5.332, 0.107]	.060
Religious affiliation	−2.532	−0.083	[−4.873, −0.190]	**.034**
Alcohol consumption	1.234	0.049	[−0.782, 3.249]	.230
Drug use	1.224	–	[−4.633, 7.080]	.682

*Note.* Standardized coefficients (β) are not reported for the categorical predictors *sex* and *drug use*, as standardized estimates are only computed for continuous variables. Bold indicate statistically significant results (*p* < 0.05).

In the second model, which examined coercive discipline, Block 1 showed significant effects of physical abuse and sexual abuse on this parenting tactic (see Table 4). After incorporating Block 2, the full model did not reach statistical significance, F (11, 688) = 1.733, p = .062, explaining only 2.7% of the variance (R^2^ = .027). However, within Block 2, educational level emerged as a significant predictor, indicating that higher educational attainment was associated with a slightly lower use of coercive discipline. No other sociodemographic variables significantly predicted this behavior (*p* > .05). The full set of coefficients is presented in Table 6.

**Table 6.** Sociodemographic Predictors of Coercive Discipline (Hierarchical Regression, Block 2)

Predictor	B	β	95% CI	p
Sex	0.230	–	[−1.655, 2.115]	.811
Age	−0.085	−0.059	[−0.197, 0.027]	.139
Marital status	−1.026	−0.066	[−2.228, 0.175]	.094
Educational level	−0.716	−0.100	[−1.302, - 0.131]	**.017**
Employment status	−0.319	−0.039	[−0.974, 0.336]	.339
Annual net income	−0.301	−0.033	[−1.135, 0.533]	.479
Nuclear family	−0.191	−0.013	[−1.284, 0.901]	.731
Ethnic group	−0.626	−0.037	[−1.977, 0.725]	.363
Religious affiliation	−0.111	−0.007	[−1.274, 1.052]	.851
Alcohol consumption	0.843	0.068	[−0.159, 1.844]	.099
Drug use	1.660	–	[−1.250, 4.569]	.263

*Note.* Standardized coefficients (β) are not reported for the categorical predictors *sex* and *drug use*, as standardized estimates are only computed for continuous variables. Bold indicate statistically significant results (*p* < 0.05).

In the third model, with physical aggression as the dependent variable, Block 1 showed significant effects of physical abuse, sexual abuse, physical neglect, and emotional neglect on this tactic (see Table 4). After incorporating Block 2, the overall model was statistically significant, F (11, 688) = 2.012, p = .025, although it explained only a small proportion of variance (R^2^ = .031). At the individual level, only two sociodemographic variables emerged as significant predictors: sex and alcohol use. Women (M = 0.27, SD = 1.79) reported lower levels of physical aggression compared to men (M = 1.79, SD = 11.12), and alcohol use was positively associated with the use of physical aggression. The remaining sociodemographic variables did not show significant effects (*p* > .05). The full coefficients for Block 2 are presented in Table 7.

**Table 7.** Sociodemographic Predictors of Physical Aggression (Hierarchical Regression, Block 2)

Predictor	B	β	95% CI	*p*
Sex	−1.801	–	[−3.056, - 0.546]	**.005**
Age	−0.034	−0.035	[−0.108, 0.041]	.377
Marital status	−0.399	−0.038	[−1.199, 0.402]	.328
Educational level	0.091	0.019	[−0.300, 0.481]	.649
Employment status	0.304	0.055	[−0.132, 0.740]	.171
Annual net income	−0.488	−0.081	[−1.044, 0.067]	.085
Nuclear family	0.285	0.029	[−0.442, 1.013]	.441
Ethnic group	−0.054	−0.005	[−0.953, 0.846]	.907
Religious affiliation	−0.113	−0.011	[−0.887, 0.661]	.775
Alcohol consumption	0.817	0.099	[0.150, 1.484]	**.016**
Drug use	0.766	–	[−1.171, 2.704]	.438

*Note.* Standardized coefficients (β) are not reported for the categorical predictors *sex* and *drug use*, as standardized estimates are only computed for continuous variables. Bold indicate statistically significant results (*p* < 0.05).

## Discussion

The main objective of this study was to analyze the presence of the cycle of violence within the Spanish context, understood as the intergenerational transmission of violent practices from parents to their sons and daughters. To this end, four objectives were addressed, which made it possible to progressively examine the associations between childhood maltreatment experiences and the later use of parental conflict tactics, as well as the moderating role of various sociodemographic variables.

Regarding the first objective, which proposed that childhood maltreatment experiences would be positively associated with greater use of aggressive parental conflict tactics (coercive discipline and physical aggression), and that no notable associations were expected with non-violent discipline, the bivariate correlation analyses revealed a consistent pattern. As expected, all forms of childhood maltreatment were positively and significantly associated with coercive discipline and physical aggression. In contrast, non-violent discipline was associated only with emotional abuse. These results support Hypothesis 1, aligning with previous research which demonstrates the link between childhood maltreatment and aggressive parental conflict tactics in adulthood (Hellmann et al., 2018; Save the Children, 2018; WHO, 2024).

With regard to the second objective, which proposed that each type of childhood maltreatment would individually predict greater use of aggressive parental conflict tactics, the simple regression analyses showed that all forms of maltreatment predicted coercive discipline. In the case of physical aggression, emotional, physical, and sexual abuse, as well as physical neglect, showed significant effects, whereas emotional neglect was not a relevant predictor. In contrast, for non-violent discipline, only emotional abuse and emotional neglect showed significant effects. This variability in the patterns of association has been previously documented, indicating that the links between childhood maltreatment experiences and parental tactics may fluctuate depending on sociocultural context, family characteristics, and other moderating factors that influence how these experiences manifest in adult parenting (Dominguez et al., 2025b; Hayes et al., 2020; Kong et al., 2021; Lakhdir et al., 2019; Ranghetti & Milani, 2021). Thus, these results partially support Hypothesis 2, showing that most forms of childhood maltreatment influence aggressive parental conflict tactics (coercive discipline and physical aggression). Nevertheless, the effect sizes observed were small to moderate—an expected pattern in psychological research, given the complexity of parental behavior and the multiplicity of individual, familial, and contextual factors involved.

Regarding the third objective, which proposed that when all forms of childhood maltreatment were considered simultaneously, the most severe types of abuse (emotional, physical, and sexual) would emerge as the main predictors of aggressive tactics, the multiple regression analyses yielded nuanced findings. For coercive discipline and physical aggression, physical abuse and sexual abuse were robust predictors. In the case of physical aggression, a positive effect of physical neglect and a negative effect of emotional neglect were also found, the latter potentially reflecting a suppression effect. Emotional abuse, however, lost significance when considered alongside the other forms of maltreatment. Overall, these findings partially support Hypothesis 3 and are consistent with previous studies documenting similar patterns (Hayes et al., 2020; Kong et al., 2021; Lakhdir et al., 2019).

In this context, although emotional abuse has widely documented psychological and social consequences —sometimes even more harmful than certain forms of physical abuse (Dominguez et al., 2025b)— in the present sample, more physical forms of abuse show a more consistent relationship with the intergenerational transmission of violence. At the same time, it is possible that emotional abuse and emotional neglect influence parental conflict tactics through different pathways, potentially even being associated in some cases with greater use of non-violent practices, perhaps as a form of emotional compensation or regulation by the parents. In other words, some individuals may actively attempt to avoid repeating the negative emotional pattern they experienced in childhood, adopting more careful or less aggressive parenting strategies. This possibility opens a particularly relevant line of research aimed at examining in greater depth how certain emotional maltreatment experiences may be linked to more nuanced —and even protective— parental responses in an effort not to replicate the harm suffered during childhood.

Given that the explained variances were moderate or low, it is reasonable to assume that multiple unmeasured factors also influence parental conflict tactics. For this reason, the fourth objective proposed that certain sociodemographic variables might exert an additional influence on parental conflict tactics, even after controlling for childhood maltreatment experiences. Overall, the results partially supported this hypothesis. Although some sociodemographic variables showed significant effects, a greater number of robust associations had been expected. It is possible that certain relationships did not emerge due to methodological limitations, as discussed later.

Regarding non-violent discipline, sex, age, and religious affiliation emerged as significant predictors. Women were more likely to employ non-violent discipline, whereas men showed a stronger tendency toward coercive and aggressive strategies—a pattern consistent with recent research on gender differences in emotion regulation and parenting practices (Martín-Ruiz et al., 2024; Rey et al., 2023; UNICEF, 2025). Age, in turn, showed a negative association with non-violent discipline. This effect can be interpreted in light of the nature of the items that constitute this construct, which describe practices commonly directed toward younger children (e.g., “explaining why the behavior was wrong,” “giving the child time to think,” “redirecting them to another activity”) (Dominguez et al., 2025b; Straus et al., 1998). As parental age increases, children are also typically older, making these strategies less appropriate or less frequently used during later developmental stages, such as adolescence. Regarding religious affiliation, some isolated effects were observed, but no stable or easily interpretable pattern emerged. For coercive discipline, educational level was the only significant predictor: individuals with lower educational attainment were more likely to use coercive tactics, consistent with prior research linking lower academic achievement to more punitive or severe parenting practices (Ahn et al., 2022; Clemens et al., 2020). Finally, in the case of physical aggression, the significant predictors were sex and alcohol use. Men showed a greater propensity to engage in aggressive parental conflict tactics, and alcohol consumption was positively associated with the use of physical aggression. These findings align with previous studies linking substance use and aggressive behaviors, particularly among men (Martín-Ruiz et al., 2024; Rey et al., 2023; UNICEF, 2025).

This study presents several limitations that should be taken into account when interpreting the findings. First, the use of a cross-sectional design and self-reported measures may introduce recall bias, particularly in the retrospective items concerning childhood maltreatment. Second, the sample shows an overrepresentation of certain sociodemographic profiles (e.g., employed individuals or those with medium income levels), which may limit the generalizability of the results. It is plausible that this sample composition affected the findings of Objective 4, reducing the likelihood of detecting broader associations between sociodemographic variables and parental conflict tactics. Along these lines, the regression models demonstrated relatively low explanatory power, suggesting that additional factors—unmeasured in the present study—may play an important role in shaping parenting practices. Nevertheless, this pattern is common in psychological research, where complex behaviors are typically influenced by multiple individual, contextual, and relational variables. For this reason, these limitations do not undermine the overall robustness of the study’s conclusions.

Future research should address these limitations through more rigorous methodological designs. Longitudinal studies would allow for the examination of temporal dynamics and causal mechanisms underlying the intergenerational transmission of violence. Moreover, recruiting more heterogeneous and socially diverse samples would help ensure a more accurate representation of the population, capturing broader variability in cultural, socioeconomic, and family contexts. It will also be essential to deepen the study of protective factors—such as emotional regulation, positive parenting competencies, and community support networks—that may buffer the impact of childhood maltreatment and promote alternative, non-violent parenting trajectories. In the same vein, the role of emotional abuse and emotional neglect warrants further investigation, as these forms of maltreatment may influence parenting through distinct mechanisms (e.g., relational sensitization, emotional repair processes). While emerging literature has begun to highlight these possibilities, more consistent empirical evidence is needed.

With regard to public policy in Spain, the findings of this study underscore the need for coordinated, trauma-informed preventive strategies at the national level. Specifically, we recommend that the Ministries of Social Rights, Equality, Health, and Education jointly develop:

(1) A systematic and unified child maltreatment screening system across pediatric services, primary education, and mental health, using validated instruments and harmonized protocols.
(2) Mandatory training in trauma-informed care, positive parenting, and intergenerational violence for healthcare, educational, and social professionals.
(3) Expanded implementation of evidence-based prevention programs in schools and community centers, prioritizing areas of higher vulnerability.
(4) Specialized trauma-informed parenting units within Child Development and Early Intervention Centers and Child and Adolescent Mental Health Centers (CDIAP and CSMIJ according to Spanish acronyms) facilities, offering trauma-focused psychological intervention, family support, and ongoing follow-up.
(5) Community-based peer-support programs for adults who experienced childhood maltreatment, designed to strengthen resilience networks and promote non-violent parenting models.
(6) Public awareness campaigns aimed at young adults —particularly future parents— highlighting the impact of childhood trauma on parenting and the resources available for support.

Together, these proposals would promote a more proactive, trauma-sensitive, and intersectoral prevention model capable of effectively interrupting the intergenerational transmission of violence. In this regard, the present study reinforces the need to integrate scientific evidence, innovation, and public policy within the Spanish context, with the ultimate goal of ensuring safe family environments and fostering healthy development for all children.

## References

[bibr1-13591045261421737] Ahn Y. Jang S. Shin J. Kim J. W. (2022). Psychological aspects of child maltreatment. The Korean Neurosurgical Society, 65(3), 408–4014. 10.3340/jkns.2021.0300 PMC9082119 35508958

[bibr2-13591045261421737] Alvarado Castillo G. J. (2025). Prevención de la transmisión de la violencia paterno-filial en contextos de violencia de género: Kintsukuroi. Educación y Futuro: Revista de Investigación Aplicada y Experiencias Educativas, 52(1), 157–191. 10.5281/zenodo.15181035

[bibr3-13591045261421737] Angelakis I. Austin J. Gooding P. (2020). Association of childhood maltreatment with suicide behaviors among young people. Jama Network, 3(8), Article 12563. 10.1001/jamanetworkopen PMC7407092 32756929

[bibr42-13591045261421737] Arfeen A. Dangwal P. (2024). Impact of childhood trauma on resilience and attachment style in adulthood. International Journal of Psychology Sciences, 6(1), 31–33. 10.33545/26648377.2024.v6.i1a.40

[bibr4-13591045261421737] Bernstein D. P. Y. Fink L. (1998). Childhood Trauma Questionnaire: A retrospective self-report (CTQ). NCS Pearson, Inc.

[bibr43-13591045261421737] Boland J Rock R Johnson A Jones M Salekin R Anderson J (2020). Pathways to incarceration: an examination of childhood maltreatment and personality psychopathology in incarcerated adults. Psychology. Crime & Law, 27(3), 253–264. 10.1080/1068316X.2020.1798426

[bibr5-13591045261421737] Bounoua N. Sadeh N. (2022). Dimensions of childhood maltreatment and adult risky behaviors: Differential affective and inhibitory control mechanisms. Child Abuse & Neglect, 134(1), Article 105877. 10.1016/j.chiabu.2022.105877 36152530 PMC12989788

[bibr7-13591045261421737] Boynton-Jarrett R. Sponholtz T. R. Rosenberg L. Palmer J. R. Bethea T. N. Wise L. A. (2021). Abuse in childhood and risk for sleep disruption in adulthood in the black women’s health study. Sleep Medicine, 83(1), 260–270. 10.1016/j.sleep.2021.02.053 34049046

[bibr8-13591045261421737] Ciurria M. (2018). The loss of autonomy in abused persons: Psychological, moral, and legal dimensions. Humanities, 7(2), 48. 10.3390/h7020048

[bibr9-13591045261421737] Clemens V. Decker O. Plener P. Witt A. Sachser C. Brähler E. Fegert J. (2020). Autohritarianism and the transgenerational transmission of corporal punishment. Child Abuse & Neglect, 106(1), Article 104537. 10.1016/j.chiabu.2020.104537 32422465

[bibr10-13591045261421737] Cohen J. (1988). Statistical power analysis for the behavioral sciences (2nd ed.). Lawrence Erlbaum Associates.

[bibr11-13591045261421737] Cuervo García A. Gracia J. (2021). El largo camino hacia la visibilidad: Un análisis victimológico de la construcción de la violencia filio-parental como problema en España. Revista de Victimología, 11(1), 21–44. 10.12827/RVJV.11.03

[bibr12-13591045261421737] Dominguez D. Pérez-Testor C. Benedico-Peydró P. Casarramona A. Aznar-Martínez B. (2025a). Assessing child maltreatment and its relationships with personality, resilience and attachment in adulthood. Children & Society, 39(4), 845–853. 10.1111/chso.12954

[bibr13-13591045261421737] Dominguez D. Pérez-Testor C. Benedico-Peydró P. Casarramona A. Aznar-Martínez B. (2025b). Spanish validation of the conflict tactics scale - Parent to child: Assessing non-violence discipline, coercive discipline, and physical aggression. Frontiers in Psychology, 16(1), 1579200. 10.3389/fpsyg.2025.1579200 40535183 PMC12175065

[bibr15-13591045261421737] Hayes B. Connolly E. Wang X. Ingham C. Mason M. (2020). Prevalence of child maltreatment and the effects of the intergenerational transmission of violence on attitudes toward domestic violence in Chinese police cadets. Journal of Family Violence, 36(6), 733–742. 10.1007/s10896-020-00182-0

[bibr16-13591045261421737] Hellmann D. Stiller A. Glaubitz C. Sörem K. (2018). (Why) do victims become perpetrators? Intergenerational transmission of parental violence in a representative German sample. Journal of Family Psychology, 32(2), 282–288. 10.1037/fam0000391 29658766

[bibr17-13591045261421737] Hernández A. Gallardo-Pujol D. Pereda N. Arntz A. Bernstein D. P. Gaviria A. M. Labad A. Valero J. Gutiérrez-Zotes J. A. (2012). Initial validation of the Spanish childhood trauma questionnaire-short form: Factor structure, reliability and association with parenting: Factor structure, reliability and association with parenting. Journal of Interpersonal Violence, 28(7), 1498–1518. 10.1177/0886260512468240 23266990

[bibr18-13591045261421737] Islam M. Broidy L. Eriksson L. Rahman M. Mazumder N. (2022). Childhood maltreatment and decision-making autonomy in adulthood: The mediating roles of self-esteem and social support. Child Abuse & Neglect, 129(1), Article 105665. 10.1016/j.chiabu.2022.105665 35567956

[bibr19-13591045261421737] Kong J. Lee H. Slack K. Lee E. (2021). The moderating role of three-generation households in the intergenerational transmission of violence. Child Abuse & Neglect, 117(1), Article 105117. 10.1016/j.chiabu.2021.105117 34022490 PMC9533149

[bibr20-13591045261421737] Lakhdir M. Nathwani A. Ali N. Faroow S. Azam S. Khaliq A. Kadir M. (2019). Interngenerational transmission of child maltreatment: Predictors of child emotional maltreatment among 11 to 17 years old children residing in communities of Karachi, Parkistan. Child Abuse & Neglect, 91(1), 109–115. 10.1016/j.chiabu.2019.03.004 30856598

[bibr23-13591045261421737] Martín-Ruiz I. García-Pérez C. Herrera-Gallego A. (2024). Ansiedad y recursos personales en la adolescencia: diferencias según sexo. European Journal of Education and Psychology, 17(1), 1–14. 10.32457/ejep.v17i1.2406

[bibr26-13591045261421737] Moya M. C. C. Rodríguez E. Q. (2025). El camino hacia una justicia más humanizadora (II): Romper el ciclo: Propuesta de una intervención psicológica obligatoria para menores expuestos a violencia de género como imperativo legal para prevenir el trauma intergeneracional en la legislación española. Diario La Ley, 10698(1), 2. https://dialnet.unirioja.es/servlet/articulo?codigo=10133179

[bibr27-13591045261421737] Ranghetti F. Milani L. (2021). Risk and protective factors regarding child neglect: Differences among immigrant and Italian parents. Journal of Aggression, Maltreatment & Trauma, 31(1), 44–54. 10.1080/10926771.2021.1970671

[bibr28-13591045261421737] Rey M. Calonge I. Martínez Arias M. R. Thomas Currás H. (2023). Sexo como variable moderadora de la sintomatología internalizante y externalizante en la infancia. Revista Española de Salud Pública, 97(1), Article e202303022. https://www.scielosp.org/article/resp/2023.v97/e202303022/36950951 PMC10558109

[bibr29-13591045261421737] Sahle B. W. Reavley N. J. Li W. Morgan A. J. Yap M. B. H. Reupert A. Jorm A. F. (2022). The association between adverse childhood experiences and common mental disorders and suicidality: An umbrella review of systematic reviews and meta-analyses. European Child & Adolescent Psychiatry, 31(10), 1489–1499. 10.1007/s00787-021-01745-2 33638709

[bibr31-13591045261421737] Save the Children . (2018). Más me duele a mí: La violencia que se ejerce en casa. https://www.savethechildren.es/sites/default/files/imce/docs/mas_me_duele_a_mi.pdf

[bibr32-13591045261421737] Selma A. A. (2022). La violencia filio-parental: Padres y madres como colectivos vulnerables en los tiempos de la Covid-19. Alternativas Político-Criminales frente al derecho penal de la aporofobia.

[bibr34-13591045261421737] Straus M. A. Hamby S. L. Finkelhor D. Moore D. W. Runyan D. (1998). Conflict tactics scale: Parent to child (CTSPC). APA PsycTests. [Data base]. 10.1037/t02127-000 9589178

[bibr35-13591045261421737] Tingtin G. Mei S. Li M. D’Arcy C. Meng X. (2021). Roles of psychological distress and social support in the relationship between childhood maltreatment and perceived needs for mental health care. Journal of Interpersonal Violence, 37(15-16), 1–28. https://doi.org/10.1177_08862605211006368 10.1177/08862605211006368 33858262

[bibr36-13591045261421737] UNICEF . (2025). El maltrato y la exposición a violencia familiar: Un estudio nacional desde la perspectiva de la adolescencia española. https://www.unicef.es/publicacion/el-maltrato-y-la-exposicion-violencia-familiar

[bibr38-13591045261421737] Warmingham J. M. Handley E. D. Russotti J. Rogosch F. A. Cicchetti D. (2021). Childhood attention problems mediate effects of child maltreatment on decision-making performance in emerging adulthood. Developmental Psychology, 57(3), 443–456. 10.1037/dev0001154 33705193 PMC8042784

[bibr40-13591045261421737] World Health Organization . (2022). Child maltreatment. https://apps.who.int/violence-info/child-maltreatment/

[bibr41-13591045261421737] World Health Organization . (2024). Maltrato infantil. https://www.who.int/es/news-room/fact-sheets/detail/child-maltreatment

